# Enhanced Selenate Removal in Aqueous Phase by Copper-Coated Activated Carbon

**DOI:** 10.3390/ma13020468

**Published:** 2020-01-19

**Authors:** Xinhai Zhao, Aiqing Zhang, Jianhong Zhang, Qipeng Wang, Xuquan Huang, Yonghong Wu, Cilai Tang

**Affiliations:** 1 College of Hydraulic & Environmental Engineering, China Three Gorges University, Yichang 443002, China; Zhao_Xh190930@163.com (X.Z.); peterwangkk@163.com (Q.W.); huangxuquan@126.com (X.H.); yhwu@issas.ac.cn (Y.W.); 2 Key Laboratory of Catalysis and Materials Science of the State Ethnic Affair Commission & Ministry of Education, College of Chemistry and Material Science, South-Central University for Nationalities, Wuhan 430074, China; aiqingzhang_2000@sina.com; 3 Resources & Environment Business Department, International Engineering Consulting Corporation, Beijing 100048, China; KZXlai@163.com; 4 State Key Laboratory of Soil and Sustainable Agriculture, Institute of Soil Sciences, Chinese Academy of Sciences, Nanjing 210008, China

**Keywords:** activated carbon, selenate, surface modification, copper, adsorption mechanism

## Abstract

In this study, we prepared a novel sorbent derived from precipitating copper ion onto the surfaces of activated carbon (Cu-AC). The sorbents were comprehensively characterized by Brunauer–Emmett–Teller (BET), zeta potential analysis, SEM, XRD, and FTIR. Batch experiments were conducted to evaluate selenate removal by Cu-AC under different conditions. The results showed that Cu was uniformly coated on the AC surface. Copper pretreatment markedly decreased the specific surface area and total pore volume of AC, and changed its surface zeta potential from highly negative to low negative and even positive. The Cu-AC substantially improved selenate adsorption capacity from the 1.36 mg Se/g AC of raw AC to 3.32, 3.56, 4.23, and 4.48 mg Se/g AC after loading of 0.1, 0.5, 1.0, and 5 mmol Cu/g AC, respectively. The results of toxicity leaching test showed AC coated with ≤1.0 mmol Cu/g was acceptable for potential application. Selenate adsorption was significantly inhibited by high ionic strength (>50 mM NaCl) and pH (>10). The electrostatic attraction between positive surface charge of Cu-AC and selenate ions and hydrogen bonding between CuO and HSeO_4_^−^ might contribute to selenate sorption. Evidence showed that the selenate adsorption might involve outer-sphere surface complexation. The adsorption data appeared to be better described by Langmuir than Freundlich isotherm. The spent adsorbent could be effectively regenerated by hydroxide for reuse. Only a little decrease of removal efficiency was observed in the second and third run. This study implies that Cu-coated AC is a potential adsorbent for sustainable removal selenate from relative low salinity water/wastewater.

## 1. Introduction

Selenium (Se) is an essential micronutrient for many living organisms but it can also be a potential toxicant to humans, livestock, and plants depending on its chemical species and dose [1,2,3]. Selenium in various forms and concentrations has been found in agricultural drainage and industrial wastewaters, such as thermoelectric power generation, mining, and refinery industries [4,5]. The increasing concerns over Se contamination from the wastewater of wet flue-gas-desulfurization (FGD) systems of coal-fired power plants have attracted more attention of the government and public [5]. The maximum level of Se in drinking water is 10 µg/L in many countries including China. Among many forms of Se, selenate (SeO_4_^2−^) is one of the most concerning forms because of its high chemical stability, mobility and bioavailability, and weak adsorption affinity to particles surface in the environment [6]. In principle, immobilization of Se can be achieved by sorption, coagulation, precipitation and ion exchanges, or by chemical and biological reduction processes [2,5,7]. Several anaerobic biological treatment processes have been developed as a solution to treat selenate contaminated mining drainage and FGD wastewater [8]. But the high costs, complexity, and other problems associated with the biological technologies are the major concerns and obstacles. Moreover, the production of more bioavailable organic Se after biological treatment is a potential harm to the environment [9]. Therefore, finding a reliable and cost-effective Se solution is still a major challenge to water industry.

Sorption is a very cost-effective and simple process for environmental contamination cleanup. Activated carbon (AC) is a very easily available and cost-effective adsorbent for contamination removal [10]. AC has been proven to be effective for adsorbing and removing a variety of organic and inorganic contaminants from aqueous and gas phase environments [11,12]. Most of studies focused on enhancing AC’s adsorption capacity for specific contaminants by modifying its surface properties via chemical, physical, or biological methods [13,14]. Activated carbon impregnated with transition metals (e.g., Fe, Al, Cu, and Ag) oxides has been used to enhance anionic contaminants removal [15,16,17]. Generally, two methods have been used to develop a metal-impregnated AC: (1) pH-induced precipitation of metal ions; and (2) adsorption of metal ions onto AC surface followed by heat treatment [17,18]. The pH method has been used more widely than the adsorption-heat treatment due to its simpler operation process and exact content of metal ion coating. It can be loaded for any amount of metal ion on AC. Generally, the adsorption of selenate onto virgin AC is very poor [18,19]. Iron, due to its relatively low environmental risk, was the most widely used transition metal to modify AC for Se removal [17,20,21]. But only selenite effective removal has been reported in previous studies by iron-impregnated AC [18,20,22]. Recently, however, copper increasingly attracted more attention as an impregnant for AC modification towards contaminants removal [23,24,25,26,27]. Copper oxide (CuO) nanoparticles impregnated AC decreased its pH_pzc_ and, thus, obviously promoted the adsorption capacity of atrazine, caffeine, diclofenac, and nitrate onto AC [26]. Hu et al. reported that copper-impregnated AC showed the best adsorption performance for ceftazidime rather than iron and aluminum [24]. Compared to Ni, Fe, and Co, Cu modified AC produced more active groups and was beneficial to activate the active component of AC, and thus exhibited the best Hg (0) removal efficiency [27]. Previous studies also showed that copper loaded AC significantly enhanced its adsorption capacity towards methylene blue [28], dyes [25,29], iodide ion [30], nitrate [26], and toxic gases (H_2_S and SO_2_) [23]. But few studies have investigated the copper-coated AC for Se sorption, especially for selenate. 

In our preliminary experiments, copper-coated AC achieved the best removal efficiency (99.9%) for selenate compared to the ACs coated with same amount (3 mmol/g AC) of iron(III), aluminum, and cobalt, which achieved 69%, 63%, and 87% removal, respectively. Moreover, this four metal ion pretreatment was better than H_2_O_2_ and HNO_3_ pretreatment, both of which only achieved 57% and 60% of selenate removal, respectively. Therefore, batch experiments were conducted to comprehensively probe selenate sorption by Cu-coated AC under different conditions. 

The objectives of this study were to: (1) evaluate selenate adsorption onto Cu-coated AC through batch experiments under different operational conditions; and (2) elucidate the mechanism(s) involved through analyses and characterization of physicochemical properties of copper-coated AC and its interactions with selenate by SEM, XRD, FTIR, Brunauer–Emmett–Teller (BET), and zeta potential measurements. 

## 2. Materials and Methods

### 2.1. Materials

All chemicals used in this study were of analytical reagent grade. The powder activated carbon was supplied by Strem Chemicals. As a pretreatment process, the AC was washed three times with de-ionized water (DI) to remove impurities, oven-dried at 105 °C for 24 h, and then stored in a plastic container for further modification. Selenate (Na_2_SeO_4_, >99.8%) stock solution was prepared using deoxygenated DI water. Dissolved oxygen (DO) was eliminated from the DI water by nitrogen gas purging and stored in an anaerobic chamber for use. Copper sulfate (CuSO_4_·4H_2_O, 99.8%) was used to prepare Cu-coated AC.

### 2.2. Preparation of Cu-Coated Activated Carbon

Fifteen (15) gram pre-cleaned AC powders were mixed with 300 mL CuSO_4_ solution of 5, 25, 50, or 250 mM in a 500 mL beaker to examine the effect of Cu concentration on selenate removal, which resulted in a mass ratio of copper to AC at 0.1, 0.5, 1.0, and 5.0 mmol Cu^2+^ per gram AC, respectively. After 5.0 min agitation, the suspension was slowly titrated with 1.0 M NaOH solution to the pH of 7.0 for complete precipitation of Cu^2+^ as Cu(OH)_2_. No aqueous Cu^2+^ was detected after NaOH treatment. The resultant suspension was filtered and then washed with DI water until SO_4_^2−^ was below <1.0 ppm in the supernatant. The washed AC was dried at 105 °C overnight. Upon cooling, the copper-coated AC was rinsed again with DI and dried at 105 °C, then comminuted and stored in plastic bottles for use. 

### 2.3. Characterization of Activated Carbon

Surface morphology of the AC before and after copper treatment was characterized using a scanning electron microscope (Quanta 600 FE-SEM, JOEL, Tokyo, Japan) at a voltage of 20 kV. The SEM is equipped with an energy dispersion spectrometer (EDS) detector that can perform elemental analysis. Specific surface area and total pore volume of the AC were analyzed with a NOVA surface area and pore size analyzer (4200e, Quantachrome Instruments, Phoenix, EDAX, Mahwah, NJ, USA) using the BET nitrogen adsorption method. Surface zeta potential was measured by the electrophoretic method using a Malvern Zetasizer Nano ZS (Phoenix, EDAX, Mahwah, NJ, USA) while ultrapure DI water was used as the dispersant without pH adjustment. X-ray diffraction (XRD) (Rigaku Ultima IV, Kyoto, Japan) using Cu *Kα* radiation was used to determine the copper compounds. Fourier transform infrared spectroscopy (FTIR) (NEXUS, Thermo Electron, Waltham, MA, USA) was used to detect the detailed properties of AC surface functional groups before and after modification.

### 2.4. Batch Experiments

The selenate adsorption experiments were conducted in serum bottles with an effective volume of 10 mL. Firstly, a predetermined amount of Cu-coated AC (e.g., 0.10 g) was placed in the serum bottles. The bottles were then transferred into an anaerobic chamber which was filled with an air of approximately 97% N_2_ and 3% H_2_ and equipped with a palladium catalytic oxygen removal system and an oxygen detector (Coy Laboratory, Mahwah, NJ, USA). Then, 10 mL diluted selenate solution was added to the bottles using pipettor, which created the initial condition of 10 g/L AC mixing with selenate of a specific concentration. The bottles were capped with a rubber stopper and sealed tightly with an aluminum crimp. Then they were transferred into a rotary shaker for complete mixing at 30 rpm at room temperature (22 ± 2 °C) in the dark. No pH adjustment was performed unless specified otherwise. At designated time interval, two bottles (duplicate) were withdrawn from the shaker and sacrificed for pH and selenate analyses after filtering with a 0.45 µm membrane filter. 

In order to evaluate the effect of initial pH on selenate adsorption, 0.5 mol HCl or NaOH mol solutions were used to adjust the initial pH of the mixed suspension to pH 4.0, 5.0, 6.0, 7.0, 8.0, 9.0, or 10.0. Ionic strength was adjusted by adding NaCl stock solution to achieve 10, 25, 50, 75, or 100 mM NaCl. Toxicity characteristic leaching procedure (TCLP) was conducted to assess copper release following the U.S. EPA TCLP method 1311 using a pH 4.93 extractant prepared from glacial acid and sodium hydroxide.

Another set of the desorption experiment was performed to evaluate the adsorbent regeneration using 1.0 mmol Cu/g AC. After reaching adsorption equilibrium, the suspension was filtered and collected filtrate for selenate measurement. The residual solid was rinsed twice with 5.0 mL DI water each time. The suspension was passed through the same filter membrane to collect all AC solid. But the filtrate was discarded. The filter membrane containing solid was then put back into previous bottle, and followed by adding 10 mL 0.10 M NaOH. The bottle was sealed tightly and transferred into the rotary shaker for complete mixing to extract adsorbed selenate. In order to investigate the effect of extracting time on the extracting efficiency of adsorbed selenate, 4, 10, 20, and 30 h were chosen as the shaking time. An extracting time of 4 h was recommended in literature to desorb selenate sorption onto iron oxides [31]. Moreover, the reuse trial of the regenerated Cu-AC was also performed similar to new adsorbent test, except that the initial suspension pH was adjusted to seven using 0.1 M HCl because NaOH was added to extract adsorbed selenate. 

### 2.5. Analytical Methods

Selenate in the filtrate was measured using a Dionex DX 500 model ion chromatographer (IC, ThermoFisher Scientific, Waltham, MA, USA) equipped with an autosampler and a CD20 conductivity detector. Separation was achieved using a Dionex IonPac AS-22 column (4 by 250 mm) and an AG-22 guard column (4 by 50 mm). The eluent solution (4.5 mM Na_2_CO_3_ and 1.4 mM NaHCO_3_) was pumped at a flow rate of 1.5 mL/min. The detection limit of the IC for selenate was 20 µg/L as Se. Dissolved copper in the leachate was also analyzed with the IC system using an IonPac CS5A 4 by 250 mm separation column and a UV-Vis detector (AD 20). The flow rate of the eluent (MetPac PDCA) and the MetPac post column reagent were 1.0mL/min and 0.5 mL/min, respectively. pH value was measured using an ORION pH meter and probe. 

## 3. Results and Discussion

### 3.1. Characteristics of the Adsorbents

**Figure 1:**
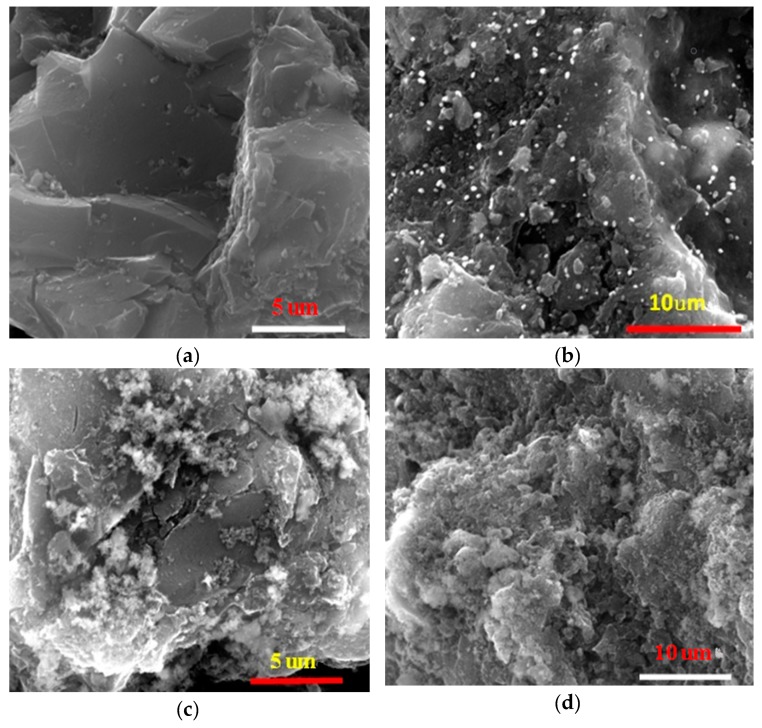
(**a**) SEM images of the raw and copper-coated activated carbon with a copper dosage of (**b**) 0.1, (**c**) 1.0, and (**d**) 5.0 mM Cu/g AC.

**Figure 2:**
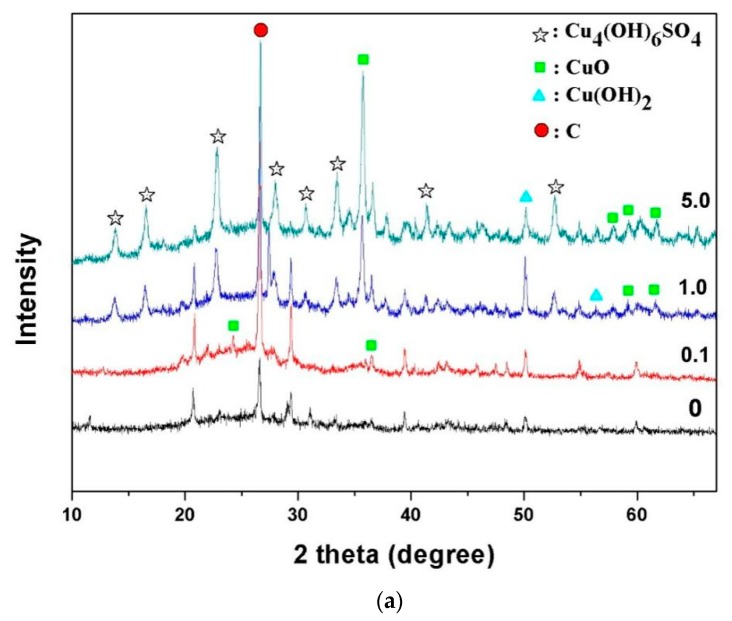

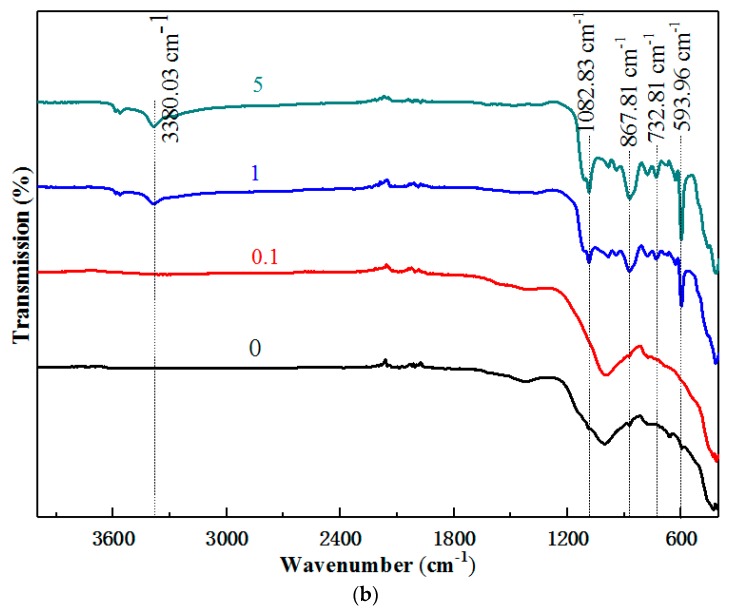
XRD patterns (**a**) and FTIR spectra (**b**) of activated carbon before and after Cu-coating with different dosage of copper loading.

As shown in Figure 1, the surface morphology of the AC particles varied a lot before and after Cu coating. The virgin AC surface was smooth, porous and rugged (Figure 1a). After Cu coating at a dosage of 0.1 mmol Cu per gram AC (Figure 1b), the AC surface was sporadically and uniformly deposited with small white particulate matters that were identified as a copper-bearing substance by EDS analysis (data not shown). The copper particles seem to be nanosized, in the range of <0.5 μm. It indicated that copper was homogeneously coated on the AC surface. More Cu-containing compound deposit on the AC surface was observed with increasing Cu dosage loading. A sectional flocculent coating at 1.0 mmol Cu/g AC (Figure 1c) and a thick and full coverage on AC surface at 5.0 mmol Cu/g AC (Figure 1d) was observed. The copper compounds form was identified using XRD (Figure 2a). Copper oxide was the dominant Cu-containing compound with 0.1 mmol Cu/g AC coating. But CuO, Cu(OH)_2_, and Cu_4_(OH)_6_SO_4_ were co-present with 1.0 and 5.0 mmol Cu/g AC coating. It implied that SO_4_^2−^ from CuSO_4_ coprecipitated with Cu(OH)_2_.

The materials were also characterized by BET specific surface area analysis (S_BET_), micropore area (S_micro_), micropore volume (V_micro_), total pore volume (V_total_), and average pore diameter (D_p_). The results are shown in Table 1. It could be seen that pure AC has a high specific surface area, and it gradually decreased with the increasing of copper coating dosage. The specific surface area of AC coated with 5 mmol Cu/g was only half of the raw AC. Similarly, the micropore area, micropore volume, and total pore volume also decreased compared to raw AC. It is possible due to the partial micropore obstruction by copper compounds, which was consistent with previous report [26]. As observed in SEM pattern in Figure 1, the copper precipitates appeared to fill up or clog a substantial portion of the micro-pores within the AC grains and thus reduce both the porosity and specific surface area of the AC. Such a decrease in pore volume and specific surface area after metal impregnation was also observed in previous studies on copper-impregnated AC [18,24,32,33,34], iron-impregnated AC [21,35], and oxidant-modified AC [36]. However, the average pore diameter increased slowly with more copper coating. Similar existence was also observed after copper modification [27]. Zeta potential is a measure of the magnitude of the electrostatic or charge repulsion/attraction between particles and is one of the fundamental parameters known to affect stability. The surface zeta potential (E_zeta_) of the raw AC was highly negative. Although the copper coating markedly decreased the negative charge of AC with increasing Cu dosage, it was still negatively charged when Cu dosage was below 1 mmol Cu/g AC. It was positively charged after coating with 1 and 5 mmol Cu/g AC. Therefore, with the higher dosage of copper coating, a higher positive of surface charge occurred. As the variation of the surface charge, the electrostatic interaction between the selenate and AC could change completely. For selenate, the interaction force between selenate and the AC would change from electrostatic repulsive to attractive and thereby significantly increase the surface affinity of selenate. Generally, the pH at point of zero charge (pH_pzc_) of activated carbon was approximately four to five, and it increased to seven to nine after modification using metal cations or metal oxides [18,26,28,32,33]. The zeta potential depends on surface charge. And the zeta potential determined the pH_pzc_. More negative zeta potential implied it needed more positive charge (e.g., H^+^) to neutralize it and its pH_pzc_ would be more acidic, vice versa. 

The surface functional groups of AC treated with different dosage of Cu loading were investigated by FTIR (Figure 2b). The small peak at around 3380 cm^−1^ was attributed to −OH stretching vibration in AC treated with 1 and 5 mmol Cu/g [37]. But no peak at this area was observed for raw AC and modified with a little of Cu loading (0.1). It was possible due to complete dehydration of Cu(OH)_2_ during drying in the presence of small dosage of Cu loading, but it was only partly dehydrated for more thick Cu coating (1 and 5 mmol). The blunt peak at around 1000 cm^−1^ was possibly attributed to sulfonic acids and salts in raw and low Cu loading AC [38], but it almost disappeared with more Cu coating. New peaks at around 1082, 867, and 594 cm^−1^ were attributed to the vibration of Cu-O after coating with more Cu loading [39,40]. Among them, the peak at 594 cm^−1^ was associated with longitudinal optical vibrational mode of Cu-O [40]. More peaks in the area of 600–1200 cm^−1^ in the Cu-AC implied that more surface specific functional groups existed after coating more Cu dosage, and thus more favorable selenate adsorption. Previous study found more peaks in the area of 800–1000 cm^−1^ after copper modification, and they proposed that copper-modified activated carbon produced more reactive atoms on the adsorbent surface and more vacant reactive metallic or semi-metallic centers, which remarkably enhanced its adsorption capacity towards methylene blue dye [25].

### 3.2. Effect of Copper Coating Dosageon Selenate Removal

**Figure 3:**
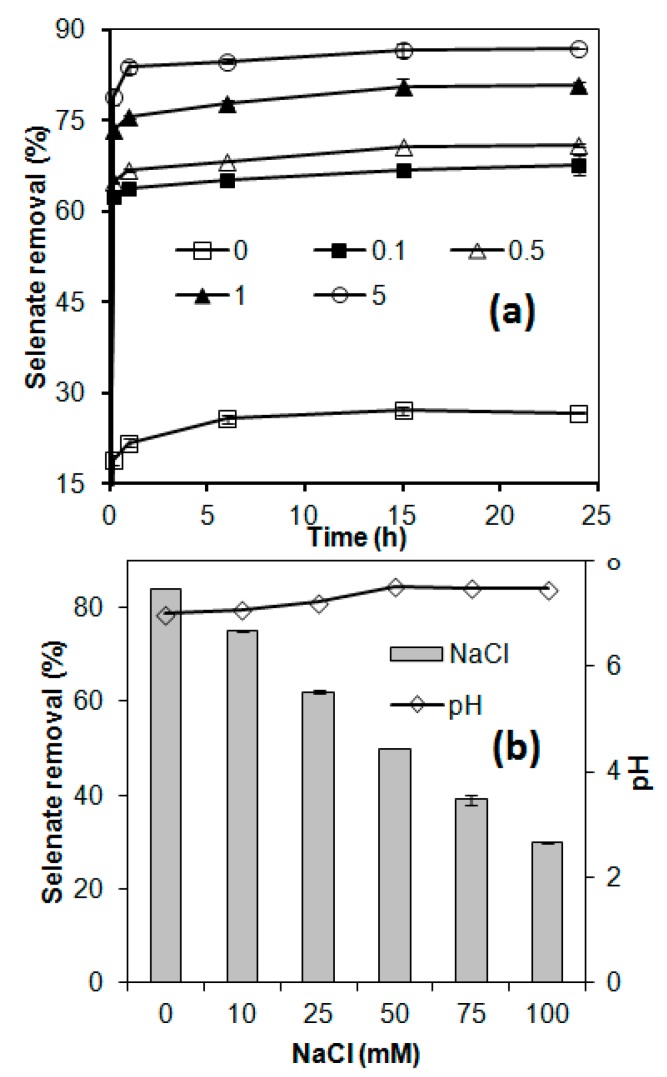
Effect of (**a**) copper coating dosage [0 (raw), 0.1, 0.5, 1.0, and 5.0 mmol Cu/g AC] on selenate adsorption; and (**b**) solution ionic strength on selenate adsorption on copper-coated activated carbon, and the solution pH after adsorption, with an initial pH ~7.0.

As shown in Figure 3a, it markedly increased selenate removal efficiency with increasing dosage of Cu coating. The raw AC could only achieve 27% selenate removal after equilibrium. In comparison, the Cu-AC achieved selenate removals of 67%, 71%, 81%, and 88% with copper dosages of 0.1, 0.5, 1.0, and 5.0 mmol/g AC, respectively. The increase in selenate removal by Cu-AC was achieved despite the decrease in the sorbent’s specific surface area and total pore volume after Cu impregnation (Table 1). The Cu treatment, however, changed the surface zeta potential from highly negative to low negative and even positive, which might be responsible for the promotion effect of adsorption capacity. This observation was in agreement with several previous studies on selenite sorption onto iron-coated AC [35], propanethiol adsorption onto copper-coated AC [33], and Cr(VI) adsorption onto HNO_3_-modified AC [36]. Compared to 10%(w/v) iron-coated AC [32], 15.6 mmol Cu/g AC [30] and 0.25–1.5 mol Cu/g AC [41], the dosage of Cu coating (0.1–5.0 mmol Cu/g AC) on AC in this study is not high. Although no dissolved copper release was detected during the whole period of sorption with all Cu-AC, the possible release of copper ion in the long-term application may pose potential risk to human and ecosystem health. Hence, the toxicity characteristic leaching procedure (TCLP, Madison, WI, America; EPA Test Method 1311) protocol on Cu-AC was conducted to assess its potential risk. The TCLP results showed that the extraction leaching contained 0.2, 4.5, 12.6, and 56.8 mg/L dissolved copper for the Cu-AC coated with 0.1, 0.5, 1.0, and 5.0 mmol Cu/g AC, respectively. The total copper content in the leachate of hazardous solid waste is 50 mg/L in China. Moreover, the soluble threshold limit concentration (STLC) of Cu is 25 mg/L in USA. Hence, AC coated with ≤1.0 mmol Cu/g is an acceptable adsorbent for potential application. The spent adsorbent can be disposed as a non-hazardous solid. In order to further evaluate the effect of other operation parameters on potential application in the future, Cu-coated AC with 1.0 mmol Cu/g AC was chosen as the adsorbent for the next experiments.

### 3.3. Effect of Ionic Strength

Generally, high ionic strength would inhibit target contaminants adsorption in aqueous phase due to competition. Many of previous studies only employed a very low salinity (≤5 mM) to probe the impact of ionic strength or co-present ion on contaminants adsorption by modified AC [35,42,43]. It is not accurate to evaluate the effect of ionic strength on adsorption, because the salinity in real water/wastewater is much higher than it. Therefore, a higher salinity (0–100 mM) was performed to investigate the effect of ionic strength on selenate adsorption onto Cu-AC in this study. The result in Figure 3b showed that ionic strength significantly inhibited the adsorption of selenate onto the Cu-AC. The increase of ionic strength reduced selenate adsorption onto the Cu-AC. Selenate removal dropped substantially from 84% to 75% when 10 mM NaCl was present. It was further reduced to 62.5%, 50.3%, 39.7%, and 30.5% when 25 mM, 50 mM, 75 mM, and 100 mM NaCl was added, respectively. The added NaCl was corresponding to the salt content of 0.59, 1.46, 2.9, 4.39, and 5.85 g/L, respectively. This salinity is comparable to most of moderate polluted water/wastewater. A small increase of pH was observed with increasing NaCl addition, possibly due to the exchange of Cl^−^ with OH^−^ on Cu-AC surface, because the presence of OH^−^ for coating 1.0 mmol Cu/g AC was proved by FTIR (Figure 2b). It showed that ionic strength not pH was the major reason for poor selenate removal. This implied that an outer-sphere complexation, which was sensitive to ionic strength, for selenate adsorption onto Cu-AC was the most possible mechanism. A previous study suggested that outer-sphere complex was a weakly-bonded affinity and could be weakened remarkably with increasing ionic strength [44]. Such negative impacts of ionic strength on contaminants adsorption was also observed in previous studies on selenate adsorption onto iron oxides/hydroxides [45], iron-coated AC [21], and iodide sorption onto Cu-AC [30]. The results suggested that the Cu-coated AC might not be effective for selenate removal from certain wastewaters containing high dissolved salts but might be effective for relative low salinity (e.g., <50 mM ionic strength) water/wastewater.

### 3.4. Effect of Initial pH

**Figure 4:**
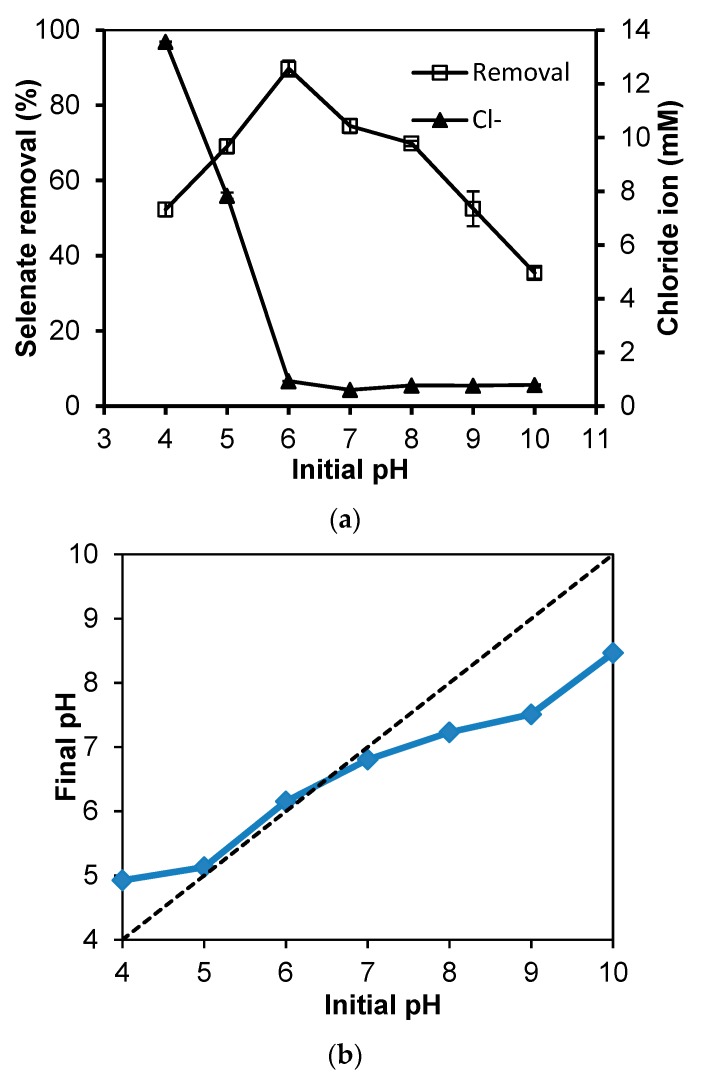
Effect of initial solution pH on (**a**) selenate adsorption onto Cu-AC, and (**b**) the final pH after adsorption.

Figure 4a shows how initial pH affected the efficiency of selenate adsorption by Cu-AC. The maximum adsorption was observed at pH 6.0. Lower or higher pH would inhibit selenate removal by Cu-AC. Previous studies also showed that selenate adsorption onto iron oxides [45], iron-coated AC [43], and ceftazidime sorption onto Cu-AC [24] decreased with increasing pH. But the lower selenate removal in the pH range of four to six might partly result from the negative effect of ionic strength as shown in Figure 3b, because 13.56 mM Cl^−^ from HCl for adjusting initial pH to 4.0 was detected. Moreover, although lower pH (4–6) might increase the positive charge of Cu-AC, it also decreases the negative charge of selenate (SeO_4_^2−^→HSeO_4_^−^), and thus it decreased their attractive force [20]. Additionally, the pH changes before and after adsorption equilibrium was obtained. As shown in Figure 4b, the pH at initial values of 4.0, 5.0, and 6.0 increased to 4.9, 5.13, and 6.15 after adsorption equilibrium, respectively. The increase of pH implied the exchange of SeO_4_^2−^ and OH^−^ on Cu-AC and the release of OH^−^. As the initial pH above 7.0, the final pH decreased possible due to OH^−^ adsorption onto Cu-AC. The similar variation of pH before and after I^−^ adsorption onto Cu-AC was observed [30].

The strong dependence of adsorption on pH and ionic strength suggested that outer-sphere complexation might be the dominant mechanism for selenate adsorption onto Cu-AC. The possible adsorption pathways could be described by the following equations:

≡Cu-OH + H^+^ + SeO_4_^2−^↔ ≡Cu-OH_2_^+^-SeO_4_^2−^ (electrostatic attraction) (1)

≡Cu-O + H^+^ + SeO_4_^2−^↔ ≡Cu-OH^+^-SeO_4_^2−^ (electrostatic attraction) (2)

≡Cu-OH + SeO_4_^2−^↔ ≡Cu-SeO_4_^−^+ OH^−^(ion exchange) (3)

≡Cu-O + H^+^ + SeO_4_^−^↔ ≡Cu-O∙∙∙∙HSeO_4_^−^ (hydrogen bond) (4)

≡Cu-OH_2_^+^ +HSeO_4_^−^↔≡Cu-OH_2_^+^-HSeO_4_^−^ (electrostatic attraction, low pH) (5)

where ≡Cu-O(H) is the reactive group on the surface of copper-coated AC. Protonation of SeO_4_^2−^ and copper-coated AC surface could be the first step under the acidic condition. Selenate was then bound onto the surface of AC by electrostatic attraction, ion exchange, and/or hydrogen bonding to form an outer-sphere complex (Equations (1)–(5)). Similar mechanisms for selenate sorption onto iron oxides [45], chromate adsorption onto iron-coated AC [46], and iodide adsorption on copper modified AC [30] have been reported in previous studies. Hydrogen bond and both monodentate and bidentate inner sphere complexes under pH < 7 and a mixture of outer sphere and inner sphere complexes at pH 8 were the possible mechanisms for selenate sorption onto iron-impregnated granular activated carbon [21].

### 3.5. Adsorption Isotherm

**Figure 5:**
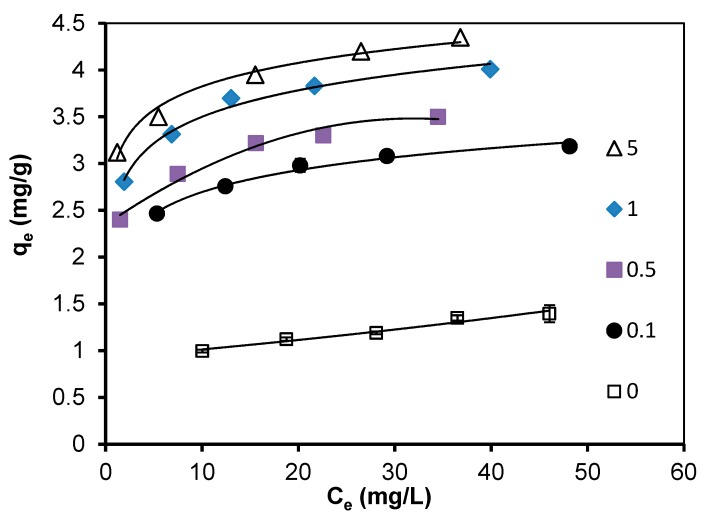
Adsorption isotherms of selenate onto activated carbon (AC) with different Cu dosage coating.

Additional adsorption experiments were conducted to probe adsorption isotherm of selenate onto AC with different Cu dosage coating (Figure 5). The results showed that a very low adsorption capacity with raw AC was obtained. It remarkably increased adsorption capacity after coating Cu. The adsorption capacity gradually increased with increasing Cu-coating dosage. Similarly, the adsorption capacity gradually increased with increasing selenate concentration.

**Figure 6:**
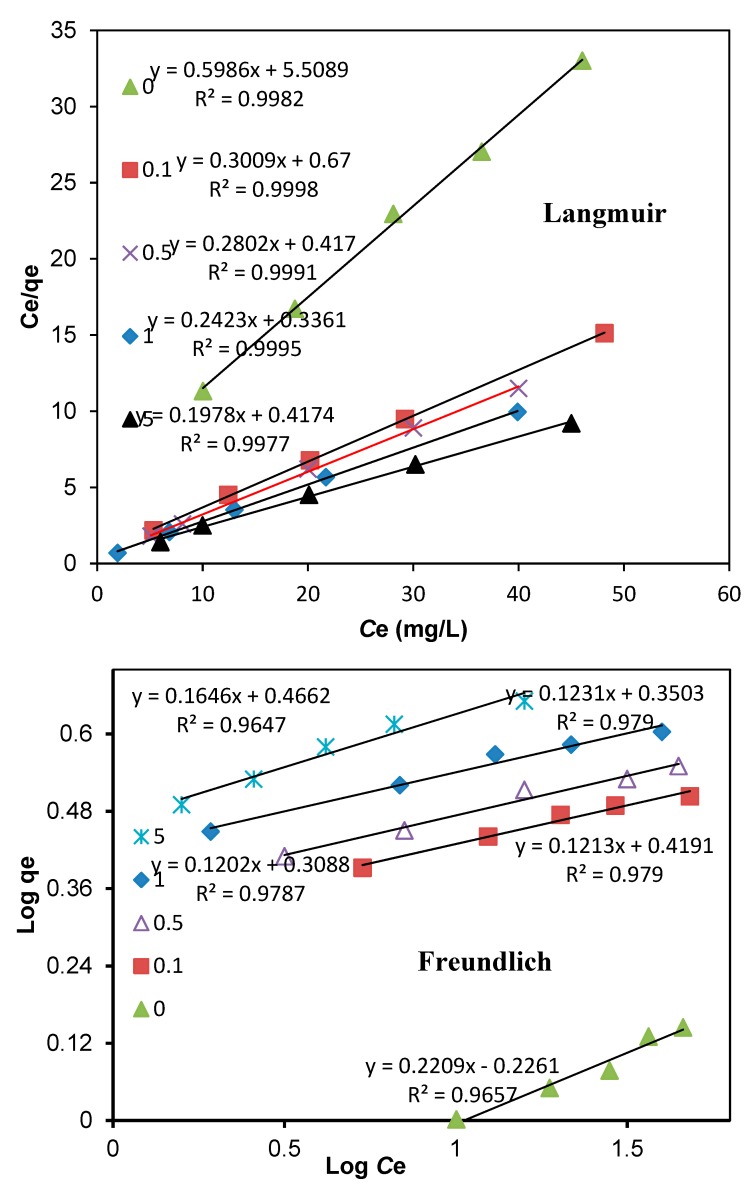
The Langmuir and Freundlich model for adsorption of selenate on copper modified AC.

Moreover, the Langmuir and Freundlich isotherms model were used to simulate the adsorption dynamics. The Langmuir model is given as below:

(6) $$q_{e}=\frac{{\mathrm{QbC}}_{e}}{1+{\mathrm{bC}}_{e}}$$

where q_e_ is the mass of selenate adsorbed per unit weight of adsorbent (mg/g), C_e_ is the equilibrium concentration of selenate in the bulk solution (mg/L), Q is saturated adsorption capacity (mg/g), and b is the constant related to the free energy of adsorption. The constants of the Langmuir isotherm, Q and b, were obtained by plotting C_e_/q_e_ versus C_e_ in a linear regression analysis (Figure 6). The Freundlich isotherm can be described as below:

(7) $$q_{e}=K_{f}C_{e}{}^{1/n}$$

where K_f_ is the constant indicative of the relative adsorption capacity of the adsorbent (mg/g), and 1/n is the constant indicative of the intensity of the adsorption. The constants, K_f_ and 1/n, were obtained by plotting log q_e_ versus C_e_ in a linear regression analysis (Figure 6).

The estimates of the Langmuir and Freundlich parameters are presented in Table 2.

According to the regression coefficients, the adsorption pattern fitted the Langmuir isotherm better than the Freundlich for all tested adsorbents (Figure 6), implying that homogeneous and monolayer sorption might be the dominant process. It was agreeable with previous study on iron-coated AC for selenite adsorption [35], copper-coated AC for propanethiol sorption [33], ceftazidime adsorption onto Cu-AC [24], and methylene blue dye sorption onto copper modified activated carbon [25]. As determined from the Langmuir isotherm, the maximum adsorption capacity (Q) for the AC modified with 0.1 mmol Cu/g AC doubled that of the raw AC. Further increase of Cu loading, however, did not proportionally increase the value of Q, and 3.56, 4.23, and 4.48 mg Se/g was obtained for coating 0.5, 1.0, and 5.0 mmol Cu/g AC, respectively. The maximum adsorption capacity of AC coating 1.0 mmol Cu/g for potential application is 4.23 mg Se/g, which is higher, in total, than selenite adsorption by iron-coated AC at 2.53–2.89 mg Se/g AC [35], selenate adsorption by iron-coated sand at 1.0 mg Se/g sand [47], and selenate sorption to iron-coated AC at 0.28–0.42 mg Se/g [43] in previous studies. The maximum adsorption capacity (*K*_f_), calculated from the Freundlich model, also showed substantial increase after Cu modification, almost quadruple from 0.585 mg/g of the raw AC to 2.036 mg/g of the treated AC with 0.1 mmol Cu. However, only a small increase in K_f_ was observed for higher copper coating AC.

### 3.6. Desorption and Reuse Study

**Figure 7:**
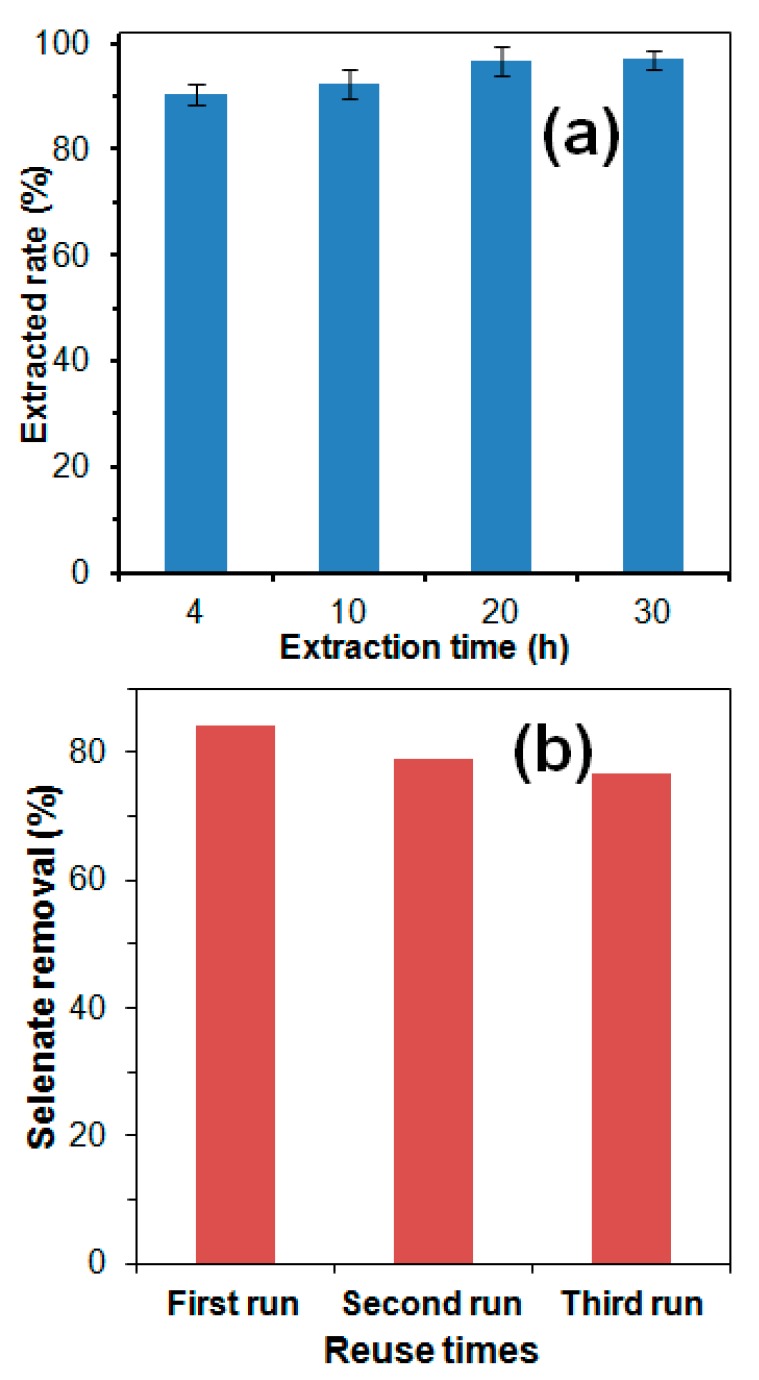
Effect of different extracted time on (**a**) adsorbed selenate desorption rate, and (**b**) the effect of reuse times on the performance of 1.0 mmol /g Cu-AC.

The reversibility of the selenate adsorption onto the Cu-AC has been further studied using 0.10 M NaOH as the desorption agent. In order to completely recover the adsorbed selenate as well as regenerate the adsorbent for reuse, 4 h recommended by literature [31], 10 h, 20 h, and 30 h of extracted time was performed. In Figure 7a, the results showed that 90.5%, 92.4%, 96.8%, and 97% of adsorbed selenate was recovered after 4, 10, 20, and 30 h extraction, respectively. A previous study showed that 4 h extraction achieved about 95% recovery of selenate adsorption onto iron oxides [28]. It indicated that selenate sorption onto Cu-AC was more tightly than onto iron oxides. Thus, for Cu-AC regeneration, 20 h extraction time is appropriate. The regenerated AC was reused for selenate sorption adjusting pH 7.0 and the results showed that 79% and 76.7% selenate removal were achieved for the second and third run, respectively (Figure 7b). The performance was close to the first run of 84%, indicating Cu-AC is a reusable adsorbent for selenate removal and thus decreases its cost. Hydroxide is an inexpensive reagent, and the regeneration operation is simple. The spent adsorbent could be regenerated for reuse, and thus it could decrease its cost in application.

## 4. Conclusions

Copper-coated AC adsorbents were prepared by simple precipitation process. The copper treatment significantly improved selenate adsorption capacity via changing the physicochemical properties of the AC surface. The modification changed the surface charge of the AC from highly negative to positive, and thus enhanced selenate adsorption through electrostatic attraction. The selenate adsorption was inhibited by high ionic strength and pH. The adsorption pattern could be described better by the Langmuir than Freundlich model. Outer-sphere complexation through electrostatic attraction, ion exchange, and hydrogen bonding could be the primary mechanism for selenate fixation on the Cu-AC. An acceptable copper leaching for the AC modified with a loading of ≤1.0 mmol Cu/g AC was observed by TCLP test on the spent sorbent. The used adsorbent could be effectively and easily regenerated by NaOH extraction for reuse. The regenerated Cu-AC could be reused three times with only a litter decrease of removal efficiency.

## Figures and Tables

**Table 1:** Textural characterization parameters and zeta potential of different sorbent.

Adsorbent	S_BET_ (m^2^/g)	S_micro_ (m^2^/g)	V_total_ (cm^3^/g)	D_p_ (nm)	E_zeta_ (mV)	V_micro_ (cm^3^/g)
0	1335	1135	0.574	1.62	−48.34	0.472
0.1	1254	1028	0.552	1.81	−32.24	0.49
0.5	1246	1002	0.536	1.79	−10.52	0.483
1.0	1132	885	0.490	1.85	5.48	0.452
5.0	679.1	475	0.326	1.92	24.80	0.274

**Table 2:** Constants of Langmuir and Freundlich equation.

Adsorbent		Langmuir			Freundlich	
(mmol Cu/g AC)	*Q*	*b*	R^2^	*K*_f_	1/*n*	R^2^
0	1.36	0.136	0.998	0.585	0.2251	0.965
0.1	3.32	0.450	0.999	2.036	0.1202	0.978
0.5	3.56	0.524	0.999	2.215	0.1208	0.979
1.0	4.23	0.721	0.999	2.625	0.1213	0.979
5.0	4.48	0.801	0.997	2.815	0.1285	0.964
